# The Fat Mass and Obesity Associated Gene, FTO, Is Also Associated with Osteoporosis Phenotypes

**DOI:** 10.1371/journal.pone.0027312

**Published:** 2011-11-18

**Authors:** Yan Guo, Hui Liu, Tie-Lin Yang, Siyang M. Li, Siyuan K. Li, Qing Tian, Yong-Jun Liu, Hong-Wen Deng

**Affiliations:** 1 Key Laboratory of Biomedical Information Engineering of Ministry of Education, and Institute of Molecular Genetics, School of Life Science and Technology, Xi'an Jiaotong University, Xi'an, People's Republic of China; 2 Department of Articular Surgery, Xi'an Red Cross Hospital, Xi'an, People's Republic of China; 3 School of Medicine, University of Missouri - Kansas City, Kansas City, Missouri, United States of America; 4 School of Public Health and Tropical Medicine, Tulane University, New Orleans, Louisiana, United States of America; 5 Center of Systematic Biomedical Research, University of Shanghai for Science and Technology, Shanghai, People's Republic of China; Leiden University Medical Center, The Netherlands

## Abstract

Obesity and osteoporosis are closely correlated genetically. *FTO* gene has been consistently identified to be associated with obesity phenotypes. A recent study reported that the mice lacking *Fto* could result in lower bone mineral density (BMD). Thus, we hypothesize that the *FTO* gene might be also important for osteoporosis phenotypes. To test for such a hypothesis, we performed an association analyses to investigate the relationship between SNPs in *FTO* and BMD at both hip and spine. A total of 141 SNPs were tested in two independent Chinese populations (818 and 809 unrelated Han subjects, respectively) and a Caucasian population (2,286 unrelated subjects). Combining the two Chinese samples, we identified 6 SNPs in *FTO* to be significantly associated with hip BMD after multiple testing adjustments, with the combined *P* values ranged from 4.99×10^−4^–1.47×10^−4^. These 6 SNPs are all located at the intron 8 of *FTO* and in high linkage disequilibrium. Each copy of the minor allele of each SNP was associated with increased hip BMD values with the effect size (beta) of ∼0.025 and ∼0.015 in the Chinese sample 1 and 2, respectively. However, none of these 6 SNPs showed significant association signal in the Caucasian sample, by presenting some extent of ethnic difference. Our findings, together with the prior biological evidence, suggest that the *FTO* gene might be a new candidate for BMD variation and osteoporosis in Chinese populations.

## Introduction

Osteoporosis is a major public health problem with growing prevalence. Obesity, another common disease, has been demonstrated to be closely related with osteoporosis [1], [2], [3]. Adipocytes and osteoblasts arise from the same progenitor, bone marrow stromal cells, and can transdifferentiate into each other [4]. With aging, the composition of bone marrow shifts to favor the presence of adipocytes, osteoclast activity increases, and osteoblast function declines, leading to osteoporosis. Moreover, both osteoporosis and obesity have high genetic predisposition and the genetic correlation between them have been established across different ethnic groups [1], [3], [5]. Previous candidate gene and bivariate association studies have suggested some common genes both for obesity and osteoporosis, such as *RANK* [6], *SP7* [7], and *SOX6* [8].

Recently, several independent large-scale genome-wide association studies (GWAS) consistently identified a gene *FTO* (fat mass and obesity associated) to be associated with obesity-related traits and obesity risk [9], [10], [11]. The association has been further replicated in multiple studies in different populations [12], [13], [14], [15], resulting in much increasing attention on this gene. The FTO protein contains a double-stranded beta-helix fold homologous to those of Fe(II) and 2-oxoglutarate-dependent oxygenases, which might be involved in DNA demethylase [16]. Experimental animal studies provide direct functional evidence that *FTO* is a causal gene underlying obesity [17], [18]. Interestingly, a recent study found that the whole body *Fto* knockout mice displayed immediate postnatal growth retardation with shorter body length, lower body weight, and lower bone mineral density (BMD) than control mice [19]. This study reminded us that *FTO* might be a common genetic factor influencing not only obesity phenotypes, but also osteoporosis phenotypes, like BMD. To test for such hypothesis, we performed an association analyses to examine the relationship between the *FTO* gene and BMD. Our study was performed in three sample sets from two ethnicities, including two Chinese Han populations and a Caucasian population, in order to see whether the variants identified are common or ethnicity-specific.

## Results

**Figure 1:**
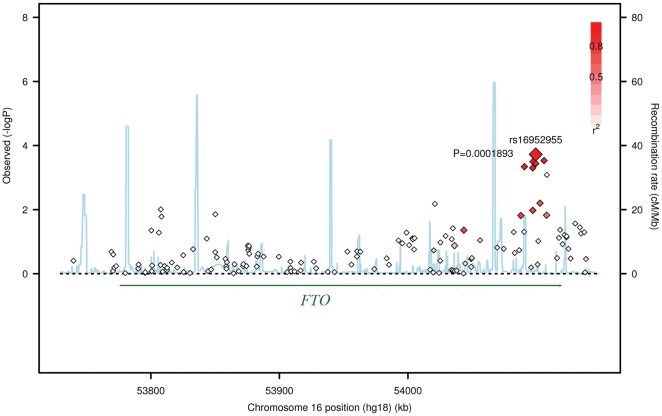
Regional Association Plot for *FTO* on chromosome 16. The color scheme of a white-to-black gradient reflects lower to higher linkage disequilibrium (LD, *r^2^*). The *r^2^* is calculated between the top significant SNP rs16952955 and other SNPs. The scatter graph indicates the negative logarithm of *P* value for each SNP, which is based on the combined results in the two Chinese samples.

The basic characteristics of the study subjects are presented in Table 1. The major association results for SNPs in *FTO* with hip BMD are summarized in Table 2. Combining results from these two Chinese samples, 6 SNPs were identified to be significantly associated with hip BMD after multiple testing adjustments by FDR (Table 2). The most significant SNP was rs16952955, with the *P* values of 8.39×10^−4^, and 4.31×10^−2^ in the Chinese sample 1 and Chinese sample 2, respectively. After meta-analysis, the combined *P* value achieved a significant level of 1.47×10^−4^. Besides rs16952955, there were 5 additional SNPs (rs2540766, rs2540784, rs16952951, rs12447427, and rs2689247) showed significant association signals with hip BMD, with the combined *P* values ranged from 4.99×10^−4^–2.95×10^−4^ (Table 2). These 6 SNPs have a consistently protective effect on BMD, since each copy of the minor allele of each SNP was associated with increased hip BMD values with the effect size (beta) of ∼0.025 and ∼0.015 in the Chinese sample 1 and 2, respectively. All of these 6 SNPs are located at the intron 8 of *FTO*. We further characterized the LD for these SNPs using the regional association plot. As shown in Figure 1, these SNPs were in high LD with the top significant SNP rs16952955 (pairwise LD *r* ^2^>0.9). For the Caucasian sample, however, no significant results were found for these 6 SNPs and the MAF of each SNP was quite different between Caucasian sample and Chinese samples (*P*<0.01 by χ^2^ test).

**Table 1:** Basic characteristics of the study subjects.

Trait	Chinese Sample 1	Chinese Sample 2	Caucasian Sample 1
Number	818	809	2,286
Female/Male	412/406	413/396	1727/558
Age (years)	28.88 (5.18)	40.18 (16.17)	51.37 (13.76)
Weight (kg)	57.37 (9.83)	62.89 (10.39)	75.27 (17.54)
Height (cm)	163.51 (7.62)	164.99 (8.62)	166.35 (8.47)
BMI (kg/m^2^)	21.38 (2.76)	23.06 (3.05)	27.14 (5.75)
Hip BMD (g/cm^2^)	0.919 (0.129)	0.921 (0.139)	0.968 (0.175)
Spine BMD (g/cm^2^)	0.960 (0.115)	0.935 (0.138)	1.025 (0.157)

Note: Data are shown as mean (standard deviation, SD).

**Table 2:** Significant association results for SNPs in *FTO* with hip BMD.

SNP	Position	Genic Position	A1/A2	Chinese Sample 1			Chinese Sample 2			*P*_combine_	Caucasian sample		
				Freq	BETA	*P*-value	Freq	BETA	*P*-value		Freq	BETA	*P*-value
rs16952955	54099469	Intron8	C/A	0.146	0.0261	8.39×10^−4^	0.132	0.0150	4.31×10^−2^	1.47×10^−4^	0.034	−0.0003	9.78×10^−1^
rs2540766	54105971	Intron8	T/C	0.144	0.0236	2.88×10^−3^	0.130	0.0165	3.25×10^−2^	2.95×10^−4^	0.042	−0.0042	6.47×10^−1^
rs2540784	54097334	Intron8	G/C	0.146	0.0239	2.38×10^−3^	0.133	0.0159	4.02×10^−2^	3.18×10^−4^	0.035	0.0006	9.52×10^−1^
rs16952951	54099427	Intron8	A/G	0.144	0.0256	1.11×10^−3^	0.132	0.0138	7.56×10^−2^	3.65×10^−4^	0.034	0.0010	9.17×10^−1^
rs12447427	54090589	Intron8	G/A	0.145	0.0241	2.03×10^−3^	0.131	0.0145	6.15×10^−2^	4.57×10^−4^	0.034	0.0005	9.61×10^−1^
rs2689247	54097159	Intron8	A/G	0.147	0.0237	2.42×10^−3^	0.132	0.0145	5.91×10^−2^	4.99×10^−4^	0.035	0.0005	9.56×10^−1^

Freq, frequency is shown for allele A1. Meta-analysis was conducted for Chinese samples 1 and 2 using the METAL software taking into account sample size and direction of effect (*P*_combine_). We listed SNPs with *P*_combine_ remaining significant after FDR adjustment.

We further performed gender-specific association analyses for the above 6 SNPs with hip BMD in the Chinese samples. The association signals in each gender group were generally weaker than in the total sample, which might be largely due to the smaller sample sizes in each gender group. The associations were mainly driven by male subjects as reflected in the Chinese sample 1, with the *P* values ranged from 0.012–1.89×10^−3^ (Table 3).

**Table 3:** Gender-specific association results for the six SNPs identified for hip BMD in the Chinese samples.

SNP	Chinese Sample 1 *P* value		Chinese Sample 2 *P* value	
	Male	Female	Male	Female
rs16952955	1.89×10^−3^	0.164	0.114	0.252
rs2540766	3.61×10^−3^	0.283	0.092	0.182
rs2540784	0.012	0.086	0.123	0.176
rs16952951	2.54×10^−3^	0.164	0.173	0.252
rs12447427	6.02×10^−3^	0.142	0.153	0.226
rs2689247	7.36×10^−3^	0.146	0.119	0.272

For the spine BMD, 5 of above 6 SNPs achieved significant level in the Chinese sample 1 (rs16952951: *P* = 8.78×10^−5^; rs16952955: *P* = 1.47×10^−4^; rs2540784: *P* = 1.97×10^−4^; rs2689247: *P* = 2.11×10^−4^; rs12447427: *P* = 4.79×10^−4^; and rs2540766: *P* = 1.99×10^−3^). However, when considering the Chinese samples 1 and 2 together, no SNPs remained significant after FDR adjustments. For the Caucasian sample, we found another 4 SNPs which are also located at the intron 8 of *FTO* to be nominally associated with spine BMD (rs11076017: *P* = 5.23×10^−3^; rs1420318: *P* = 6.14×10^−3^; rs1876942: *P* = 7.21×10^−3^; and rs17226942: *P* = 9.23×10^−3^).

For the previously reported SNP rs9939609 identified by GWAS for obesity phenotypes [9], [10], [11], we only detected nominally significant association with spine BMD in the Chinese sample 1 (*P* = 0.037).

## Discussion

The *FTO* gene has become the hotspot for researchers since it was reported as a novel obesity-susceptibility gene by a number of GWAS and follow-up replication studies. Given the evidence that obesity and osteoporosis share some common genetic determinations, we first performed an association study examining the SNPs in *FTO* for association with BMD. We identified a cluster of SNPs in *FTO* to be significantly associated with hip BMD in Chinese populations.

The human *FTO* gene is expressed in many tissues including mesenteric fat, pancreas, liver and adipose tissue, with the highest concentrations found in the hypothalamus [10], [20]. Functional studies have demonstrated the direct effect of *FTO* on obesity. For example, Fischer et al. have reported that the loss of *Fto* in mice leads to postnatal growth retardation and a significant reduction in adipose tissue and lean body mass [18]. Church et al. have shown that a mutation in the mouse *Fto* gene results in reduced fat mass, increased energy expenditure, and unchanged physical activity [17]. In respect to *FTO*'s relevance to osteoporosis, a recent study by Gao et al. has found that *Fto* plays an essential role in postnatal growth. The mice lacking *Fto* completely displayed postnatal growth retardation manifested as reduced body weight and length, lower BMD [19]. Taking into account of this biological evidence and our statistical findings, we suggest that *FTO* might have potential role on BMD or osteoporosis.

It is necessary to examine the associations in different populations from different ethnicities, since the genomic variation is greater when compared across ethnicities. Our study identified consistent association for 6 SNPs in intron 8 of *FTO* with hip BMD in two Chinese populations. Unfortunately, such results were not replicated in the Caucasian population, which implied some extent of ethnic difference. Such ethnic difference could be explained from two aspects. First, the allele frequencies are quite different between the Chinese and Caucasian populations. The minor alleles of these 6 significant SNPs were much common in the Chinese than in the Caucasians, which may contribute to the overall effect. Second, the difference might be age-specific. The two Chinese populations were relatively younger than the Caucasians. Although we have included age as a covariate to adjust BMD, it could not eliminate the potential confounding effect of age on BMD variations thoroughly. Since animal studies have reported that Fto plays an important role in postnatal growth [19], this might suggest that the *FTO* gene might be a candidate genetic marker for peak bone mass acquisition. However, it is still too early to get the conclusion. We found several other SNPs which are also in intron 8 of *FTO* for nominally significant association with spine BMD in the Caucasian population, but not in the Chinese populations. Additional studies are needed to investigate the true effect of *FTO* on BMD in Caucasian populations.

The association of *FTO* with spine BMD was less significant than with hip BMD in our study, since we only observed significant associations between *FTO* and spine BMD in the Chinese sample 1. When considering the two Chinese samples together, no SNPs remained significant after multiple testing adjustments. Our results suggest that the effect of *FTO* on hip BMD might be stronger than spine BMD. However, it is still too soon to get such conclusion. The sample size of the Chinese samples was relatively small, which might decrease the statistical power to detect genetic associations. Increasing the sample size in further studies is needed to validate our results, and we are hopeful that publication of our findings will facilitate replication analyses by other groups.

In summary, our data provide novel evidence that a cluster of SNPs in *FTO* are associated BMD variations in Chinese populations. Considering the prior biological findings, we suggest that *FTO* might be a new candidate for osteoporosis. Further studies are warranted to explore the generality of our findings and elucidate the true functional variant.

## Materials and Methods

### Subjects

The study was approved by the Institutional Review Board or Research Administration of Xi'an Jiaotong University, Hunan Normal University, Creighton University and University of Missouri-Kansas City. Signed informed consent documents were obtained from all study participants before entering the study.

#### Chinese samples

The Chinese sample 1 comprised 818 unrelated subjects, which were recruited from Southwest Chinese Han adults living in Changsha city and its neighboring areas. The Chinese sample 2 comprised 809 unrelated subjects drawing from Northwest Chinese Han adults in Xi'an City and its neighboring areas. For all the subjects, the exclusion criteria have been detailed in our earlier publication [21]. Briefly, subjects with chronic diseases and conditions that might potentially affect bone mass, structure, or metabolism were excluded from the study to minimize the influence of known environmental and therapeutic factors on bone variation. BMD at hip and spine was measured using Hologic 4500 W machines (Hologic Inc., Bedford, MA, USA) under the same strict protocols. The coefficient of variation (CV) values of the dual-energy X-ray absorptionmetry (DXA) measurements for spine and hip BMDs were approximately 1.01% and 1.33%, respectively.

#### Caucasian sample

The Caucasian sample consisted of 2,286 unrelated adults. All of the subjects were US Caucasians of Northern European origin living in Midwestern area. The exclusion criteria were the same as with Chinese samples. BMD at hip and spine were measured using the same model Hologic 4500 W machines (Hologic Inc., Bedford, MA, USA) under the same strict protocols used in the Chinese sample. The CV values of the DXA measurements for spine and hip BMDs were approximately 1.98% and 1.87%, respectively.

### Genotyping

Genomic DNA was extracted from peripheral blood leukocytes using standard protocols. For all the three samples, SNP genotyping was performed using Genome-Wide Human SNP Array 6.0 (Affymetrix, Santa Clara, CA, USA), according to the Affymetrix protocol. Only samples with a minimum call rate of 95% were included. Due to efforts of repeat experiments, all samples met this criteria and the final mean call rate reached a high level of 98.93% for Caucasian sample and 98.96% for Chinese sample. SNPs that deviated from Hardy-Weinberg equilibrium (HWE, *P*<0.0001) and had a minor allele frequency (MAF)<0.01 were discarded in each sample set. Thus, 141 SNPs in *FTO* were included for subsequent association analyses. The basic characteristics of these SNPs are summarized in Table S1.

### Statistical analyses

Before association analyses, principal component analysis implemented in EIGENSTRAT [22] was used to adjust for potential population stratification that may lead to spurious association results. The first ten principal components emerging from the EIGENSTRAT analyses, along with sex, age, height, weight and BMI, were used as covariates to adjust the raw BMD values in each sample. The residues were used for association analyses. Linear regression implemented in PLINK [23] was used to test for association under the additive inheritance model.

Meta-analysis statistics were generated using METAL software package (http://genome.sph.umich.edu/wiki/METAL_Documentation) taking into account sample size and direction of effect. SNAP was used to characterize linkage disequilibrium (LD) and depict the regional association plot [24]. A raw *P* value of <0.05 in our study was considered nominally significant, which were further subjected to a false discovery rate (FDR) of Benjamini and Hochberg procedure [25] to account for multiple comparisons.

## Supporting Information

Table S1 Properties of *FTO* SNPs tested in this study
(DOC)
