# Supplementary material for: The Fat Mass and Obesity Associated Gene, FTO, Is Also Associated with Osteoporosis Phenotypes
Source: PLoS One. 2011 Nov 18;6(11):e27312. doi: 10.1371/journal.pone.0027312 (PMC3220685; doi:10.1371/journal.pone.0027312)
Supplement: Table S1 — Properties of FTO SNPs tested in this study (DOC) [file pone.0027312.s001.doc]

Table S1. Properties of *FTO* SNPs tested in this study

| SNP | NCBI_Position | Genic Position | Allele 1 | Allele 2 |
| --- | --- | --- | --- | --- |
| rs1421091 | [53739773](http://www.ncbi.nlm.nih.gov/nuccore/NT_010498.15?report=graph&m=53739773&v=53739723:53739823&c=3366FF&theme=Details&flip=false&select=null&content=5&color=0&decor=0&layout=0&spacing=0) | Intron1 | G | T |
| rs7203521 | [53769293](http://www.ncbi.nlm.nih.gov/nuccore/NT_010498.15?report=graph&m=53769293&v=53769243:53769343&c=3366FF&theme=Details&flip=false&select=null&content=5&color=0&decor=0&layout=0&spacing=0) | Intron1 | A | G |
| rs16952479 | [53770578](http://www.ncbi.nlm.nih.gov/nuccore/NT_010498.15?report=graph&m=53770578&v=53770528:53770628&c=3366FF&theme=Details&flip=false&select=null&content=5&color=0&decor=0&layout=0&spacing=0) | Intron1 | A | T |
| rs8048396 | [53770749](http://www.ncbi.nlm.nih.gov/nuccore/NT_010498.15?report=graph&m=53770749&v=53770699:53770799&c=3366FF&theme=Details&flip=false&select=null&content=5&color=0&decor=0&layout=0&spacing=0) | Intron1 | A | C |
| rs16952482 | [53771583](http://www.ncbi.nlm.nih.gov/nuccore/NT_010498.15?report=graph&m=53771583&v=53771533:53771633&c=3366FF&theme=Details&flip=false&select=null&content=5&color=0&decor=0&layout=0&spacing=0) | Intron1 | C | T |
| rs4396532 | [53773047](http://www.ncbi.nlm.nih.gov/nuccore/NT_010498.15?report=graph&m=53773047&v=53772997:53773097&c=3366FF&theme=Details&flip=false&select=null&content=5&color=0&decor=0&layout=0&spacing=0) | Intron1 | A | G |
| rs7186637 | [53780102](http://www.ncbi.nlm.nih.gov/nuccore/NT_010498.15?report=graph&m=53780102&v=53780052:53780152&c=3366FF&theme=Details&flip=false&select=null&content=5&color=0&decor=0&layout=0&spacing=0) | Intron1 | C | T |
| rs1861869 | [53790181](http://www.ncbi.nlm.nih.gov/nuccore/NT_010498.15?report=graph&m=53790181&v=53790131:53790231&c=3366FF&theme=Details&flip=false&select=null&content=5&color=0&decor=0&layout=0&spacing=0) | Intron1 | C | G |
| rs1861868 | [53790402](http://www.ncbi.nlm.nih.gov/nuccore/NT_010498.15?report=graph&m=53790402&v=53790352:53790452&c=3366FF&theme=Details&flip=false&select=null&content=5&color=0&decor=0&layout=0&spacing=0) | Intron1 | C | T |
| rs9940700 | [53795409](http://www.ncbi.nlm.nih.gov/nuccore/NT_010498.15?report=graph&m=53795409&v=53795359:53795459&c=3366FF&theme=Details&flip=false&select=null&content=5&color=0&decor=0&layout=0&spacing=0) | Intron1 | C | G |
| rs13334933 | [53795636](http://www.ncbi.nlm.nih.gov/nuccore/NT_010498.15?report=graph&m=53795636&v=53795586:53795686&c=3366FF&theme=Details&flip=false&select=null&content=5&color=0&decor=0&layout=0&spacing=0) | Intron1 | A | G |
| rs12446228 | [53800387](http://www.ncbi.nlm.nih.gov/nuccore/NT_010498.15?report=graph&m=53800387&v=53800337:53800437&c=3366FF&theme=Details&flip=false&select=null&content=5&color=0&decor=0&layout=0&spacing=0) | Intron1 | A | G |
| rs9939973 | [53800568](http://www.ncbi.nlm.nih.gov/nuccore/NT_010498.15?report=graph&m=53800568&v=53800518:53800618&c=3366FF&theme=Details&flip=false&select=null&content=5&color=0&decor=0&layout=0&spacing=0) | Intron1 | A | G |
| rs9940128 | [53800754](http://www.ncbi.nlm.nih.gov/nuccore/NT_010498.15?report=graph&m=53800754&v=53800704:53800804&c=3366FF&theme=Details&flip=false&select=null&content=5&color=0&decor=0&layout=0&spacing=0) | Intron1 | A | G |
| rs1421085 | 53800954 | Intron1 | C | T |
| rs9922047 | 53806280 | Intron1 | C | G |
| rs16952522 | 53807498 | Intron1 | C | G |
| rs17817288 | 53807764 | Intron1 | A | G |
| rs1477196 | 53808258 | Intron1 | A | G |
| rs1121980 | 53809247 | Intron1 | A | G |
| rs7193144 | 53810686 | Intron1 | C | T |
| rs16945088 | 53812524 | Intron1 | A | G |
| rs8057044 | 53812614 | Intron1 | A | G |
| rs8050136 | 53816275 | Intron1 | A | C |
| rs9939609 | 53820527 | Intron1 | A | T |
| rs9931164 | [53825238](http://www.ncbi.nlm.nih.gov/nuccore/NT_010498.15?report=graph&m=53825238&v=53825188:53825288&c=3366FF&theme=Details&flip=false&select=null&content=5&color=0&decor=0&layout=0&spacing=0) | Intron1 | A | G |
| rs9941349 | [53825488](http://www.ncbi.nlm.nih.gov/nuccore/NT_010498.15?report=graph&m=53825488&v=53825438:53825538&c=3366FF&theme=Details&flip=false&select=null&content=5&color=0&decor=0&layout=0&spacing=0) | Intron1 | C | T |
| rs9930506 | [53830465](http://www.ncbi.nlm.nih.gov/nuccore/NT_010498.15?report=graph&m=53830465&v=53830415:53830515&c=3366FF&theme=Details&flip=false&select=null&content=5&color=0&decor=0&layout=0&spacing=0) | Intron1 | A | G |
| rs2111650 | [53832816](http://www.ncbi.nlm.nih.gov/nuccore/NT_010498.15?report=graph&m=53832816&v=53832766:53832866&c=3366FF&theme=Details&flip=false&select=null&content=5&color=0&decor=0&layout=0&spacing=0) | Intron1 | A | G |
| rs6499646 | [53843533](http://www.ncbi.nlm.nih.gov/nuccore/NT_010498.15?report=graph&m=53843533&v=53843483:53843583&c=3366FF&theme=Details&flip=false&select=null&content=5&color=0&decor=0&layout=0&spacing=0) | Intron1 | C | T |
| rs17218700 | [53844579](http://www.ncbi.nlm.nih.gov/nuccore/NT_010498.15?report=graph&m=53844579&v=53844529:53844629&c=3366FF&theme=Details&flip=false&select=null&content=5&color=0&decor=0&layout=0&spacing=0) | Intron2 | A | G |
| rs9935403 | [53846926](http://www.ncbi.nlm.nih.gov/nuccore/NT_010498.15?report=graph&m=53846926&v=53846876:53846976&c=3366FF&theme=Details&flip=false&select=null&content=5&color=0&decor=0&layout=0&spacing=0) | Intron2 | A | G |
| rs11075994 | [53850079](http://www.ncbi.nlm.nih.gov/nuccore/NT_010498.15?report=graph&m=53850079&v=53850029:53850129&c=3366FF&theme=Details&flip=false&select=null&content=5&color=0&decor=0&layout=0&spacing=0) | Intron2 | A | G |
| rs1421090 | [53850170](http://www.ncbi.nlm.nih.gov/nuccore/NT_010498.15?report=graph&m=53850170&v=53850120:53850220&c=3366FF&theme=Details&flip=false&select=null&content=5&color=0&decor=0&layout=0&spacing=0) | Intron2 | A | G |
| rs9972717 | [53851304](http://www.ncbi.nlm.nih.gov/nuccore/NT_010498.15?report=graph&m=53851304&v=53851254:53851354&c=3366FF&theme=Details&flip=false&select=null&content=5&color=0&decor=0&layout=0&spacing=0) | Intron2 | A | G |
| rs11075996 | [53858024](http://www.ncbi.nlm.nih.gov/nuccore/NT_010498.15?report=graph&m=53858024&v=53857974:53858074&c=3366FF&theme=Details&flip=false&select=null&content=5&color=0&decor=0&layout=0&spacing=0) | Intron2 | C | T |
| rs2042032 | [53858583](http://www.ncbi.nlm.nih.gov/nuccore/NT_010498.15?report=graph&m=53858583&v=53858533:53858633&c=3366FF&theme=Details&flip=false&select=null&content=5&color=0&decor=0&layout=0&spacing=0) | Intron2 | A | G |
| rs10852522 | [53858777](http://www.ncbi.nlm.nih.gov/nuccore/NT_010498.15?report=graph&m=53858777&v=53858727:53858827&c=3366FF&theme=Details&flip=false&select=null&content=5&color=0&decor=0&layout=0&spacing=0) | Intron2 | A | T |
| rs7195539 | [53859158](http://www.ncbi.nlm.nih.gov/nuccore/NT_010498.15?report=graph&m=53859158&v=53859108:53859208&c=3366FF&theme=Details&flip=false&select=null&content=5&color=0&decor=0&layout=0&spacing=0) | Intron2 | A | G |
| rs7204916 | [53864400](http://www.ncbi.nlm.nih.gov/nuccore/NT_010498.15?report=graph&m=53864400&v=53864350:53864450&c=3366FF&theme=Details&flip=false&select=null&content=5&color=0&decor=0&layout=0&spacing=0) | Intron3 | C | T |
| rs10521308 | [53865126](http://www.ncbi.nlm.nih.gov/nuccore/NT_010498.15?report=graph&m=53865126&v=53865076:53865176&c=3366FF&theme=Details&flip=false&select=null&content=5&color=0&decor=0&layout=0&spacing=0) | Intron3 | A | G |
| rs16952577 | [53868316](http://www.ncbi.nlm.nih.gov/nuccore/NT_010498.15?report=graph&m=53868316&v=53868266:53868366&c=3366FF&theme=Details&flip=false&select=null&content=5&color=0&decor=0&layout=0&spacing=0) | Intron3 | G | T |
| rs17818902 | [53871806](http://www.ncbi.nlm.nih.gov/nuccore/NT_010498.15?report=graph&m=53871806&v=53871756:53871856&c=3366FF&theme=Details&flip=false&select=null&content=5&color=0&decor=0&layout=0&spacing=0) | Intron3 | G | T |
| rs17818920 | [53871903](http://www.ncbi.nlm.nih.gov/nuccore/NT_010498.15?report=graph&m=53871903&v=53871853:53871953&c=3366FF&theme=Details&flip=false&select=null&content=5&color=0&decor=0&layout=0&spacing=0) | Intron3 | A | C |
| rs6499651 | [53874401](http://www.ncbi.nlm.nih.gov/nuccore/NT_010498.15?report=graph&m=53874401&v=53874351:53874451&c=3366FF&theme=Details&flip=false&select=null&content=5&color=0&decor=0&layout=0&spacing=0) | Intron3 | C | T |
| rs8053367 | [53875484](http://www.ncbi.nlm.nih.gov/nuccore/NT_010498.15?report=graph&m=53875484&v=53875434:53875534&c=3366FF&theme=Details&flip=false&select=null&content=5&color=0&decor=0&layout=0&spacing=0) | Intron3 | G | T |
| rs8053740 | [53875712](http://www.ncbi.nlm.nih.gov/nuccore/NT_010498.15?report=graph&m=53875712&v=53875662:53875762&c=3366FF&theme=Details&flip=false&select=null&content=5&color=0&decor=0&layout=0&spacing=0) | Intron3 | C | G |
| rs7203051 | [53876149](http://www.ncbi.nlm.nih.gov/nuccore/NT_010498.15?report=graph&m=53876149&v=53876099:53876199&c=3366FF&theme=Details&flip=false&select=null&content=5&color=0&decor=0&layout=0&spacing=0) | Intron3 | C | G |
| rs7205009 | [53876444](http://www.ncbi.nlm.nih.gov/nuccore/NT_010498.15?report=graph&m=53876444&v=53876394:53876494&c=3366FF&theme=Details&flip=false&select=null&content=5&color=0&decor=0&layout=0&spacing=0) | Intron3 | C | T |
| rs7205213 | [53876566](http://www.ncbi.nlm.nih.gov/nuccore/NT_010498.15?report=graph&m=53876566&v=53876516:53876616&c=3366FF&theme=Details&flip=false&select=null&content=5&color=0&decor=0&layout=0&spacing=0) | Intron3 | C | T |
| rs8061228 | [53882371](http://www.ncbi.nlm.nih.gov/nuccore/NT_010498.15?report=graph&m=53882371&v=53882321:53882421&c=3366FF&theme=Details&flip=false&select=null&content=5&color=0&decor=0&layout=0&spacing=0) | Intron4 | C | T |
| rs11075999 | [53882859](http://www.ncbi.nlm.nih.gov/nuccore/NT_010498.15?report=graph&m=53882859&v=53882809:53882909&c=3366FF&theme=Details&flip=false&select=null&content=5&color=0&decor=0&layout=0&spacing=0) | Intron4 | A | C |
| rs2111114 | [53883452](http://www.ncbi.nlm.nih.gov/nuccore/NT_010498.15?report=graph&m=53883452&v=53883402:53883502&c=3366FF&theme=Details&flip=false&select=null&content=5&color=0&decor=0&layout=0&spacing=0) | Intron4 | C | T |
| rs12597422 | [53887738](http://www.ncbi.nlm.nih.gov/nuccore/NT_010498.15?report=graph&m=53887738&v=53887688:53887788&c=3366FF&theme=Details&flip=false&select=null&content=5&color=0&decor=0&layout=0&spacing=0) | Intron4 | A | G |
| rs9936768 | 53899563 | Intron4 | C | T |
| rs8060649 | [53906099](http://www.ncbi.nlm.nih.gov/nuccore/NT_010498.15?report=graph&m=53906099&v=53906049:53906149&c=3366FF&theme=Details&flip=false&select=null&content=5&color=0&decor=0&layout=0&spacing=0) | Intron4 | A | G |
| rs8053707 | [53908603](http://www.ncbi.nlm.nih.gov/nuccore/NT_010498.15?report=graph&m=53908603&v=53908553:53908653&c=3366FF&theme=Details&flip=false&select=null&content=5&color=0&decor=0&layout=0&spacing=0) | Intron5 | C | T |
| rs10521304 | [53908657](http://www.ncbi.nlm.nih.gov/nuccore/NT_010498.15?report=graph&m=53908657&v=53908607:53908707&c=3366FF&theme=Details&flip=false&select=null&content=5&color=0&decor=0&layout=0&spacing=0) | Intron5 | C | T |
| rs10521303 | [53909185](http://www.ncbi.nlm.nih.gov/nuccore/NT_010498.15?report=graph&m=53909185&v=53909135:53909235&c=3366FF&theme=Details&flip=false&select=null&content=5&color=0&decor=0&layout=0&spacing=0) | Intron5 | G | T |
| rs1362571 | [53911770](http://www.ncbi.nlm.nih.gov/nuccore/NT_010498.15?report=graph&m=53911770&v=53911720:53911820&c=3366FF&theme=Details&flip=false&select=null&content=5&color=0&decor=0&layout=0&spacing=0) | Intron5 | G | T |
| rs1558756 | [53916508](http://www.ncbi.nlm.nih.gov/nuccore/NT_010498.15?report=graph&m=53916508&v=53916458:53916558&c=3366FF&theme=Details&flip=false&select=null&content=5&color=0&decor=0&layout=0&spacing=0) | Intron6 | A | G |
| rs9934504 | [53916879](http://www.ncbi.nlm.nih.gov/nuccore/NT_010498.15?report=graph&m=53916879&v=53916829:53916929&c=3366FF&theme=Details&flip=false&select=null&content=5&color=0&decor=0&layout=0&spacing=0) | Intron6 | A | G |
| rs17820875 | [53926790](http://www.ncbi.nlm.nih.gov/nuccore/NT_010498.15?report=graph&m=53926790&v=53926740:53926840&c=3366FF&theme=Details&flip=false&select=null&content=5&color=0&decor=0&layout=0&spacing=0) | Intron7 | A | G |
| rs9926180 | [53928607](http://www.ncbi.nlm.nih.gov/nuccore/NT_010498.15?report=graph&m=53928607&v=53928557:53928657&c=3366FF&theme=Details&flip=false&select=null&content=5&color=0&decor=0&layout=0&spacing=0) | Intron7 | A | T |
| rs2111112 | [53937632](http://www.ncbi.nlm.nih.gov/nuccore/NT_010498.15?report=graph&m=53937632&v=53937582:53937682&c=3366FF&theme=Details&flip=false&select=null&content=5&color=0&decor=0&layout=0&spacing=0) | Intron7 | C | T |
| rs12935710 | [53942805](http://www.ncbi.nlm.nih.gov/nuccore/NT_010498.15?report=graph&m=53942805&v=53942755:53942855&c=3366FF&theme=Details&flip=false&select=null&content=5&color=0&decor=0&layout=0&spacing=0) | Intron7 | C | T |
| rs1344503 | [53952946](http://www.ncbi.nlm.nih.gov/nuccore/NT_010498.15?report=graph&m=53952946&v=53952896:53952996&c=3366FF&theme=Details&flip=false&select=null&content=5&color=0&decor=0&layout=0&spacing=0) | Intron7 | A | G |
| rs16952649 | [53956052](http://www.ncbi.nlm.nih.gov/nuccore/NT_010498.15?report=graph&m=53956052&v=53956002:53956102&c=3366FF&theme=Details&flip=false&select=null&content=5&color=0&decor=0&layout=0&spacing=0) | Intron7 | C | T |
| rs12918495 | [53960365](http://www.ncbi.nlm.nih.gov/nuccore/NT_010498.15?report=graph&m=53960365&v=53960315:53960415&c=3366FF&theme=Details&flip=false&select=null&content=5&color=0&decor=0&layout=0&spacing=0) | Intron7 | C | T |
| rs12932428 | 53960527 | Intron7 | C | T |
| rs11864972 | 53963063 | Intron7 | A | G |
| rs7205426 | 53973807 | Intron8 | A | C |
| rs7203181 | 53983480 | Intron8 | A | C |
| rs12925189 | 53985273 | Intron8 | A | G |
| rs6499658 | 53992704 | Intron8 | A | T |
| rs11644943 | 53995584 | Intron8 | A | T |
| rs17823199 | 53998930 | Intron8 | C | T |
| rs1111483 | 54000907 | Intron8 | A | C |
| rs7194907 | 54003483 | Intron8 | C | T |
| rs8053888 | 54003805 | Intron8 | C | T |
| rs9940629 | 54004811 | Intron8 | A | G |
| rs9932411 | 54005163 | Intron8 | C | T |
| rs13337356 | 54017310 | Intron8 | C | G |
| rs12324955 | 54019686 | Intron8 | A | G |
| rs1125392 | 54020145 | Intron8 | C | T |
| rs8049235 | 54021009 | Intron8 | A | G |
| rs4784337 | 54024266 | Intron8 | A | G |
| rs8056199 | 54025172 | Intron8 | A | G |
| rs8056502 | 54025314 | Intron8 | C | G |
| rs11864881 | 54029304 | Intron8 | A | C |
| rs1861356 | 54033845 | Intron8 | C | T |
| rs7205987 | 54034145 | Intron8 | C | T |
| rs13334214 | 54034941 | Intron8 | C | T |
| rs16952770 | 54034952 | Intron8 | C | T |
| rs13335453 | 54036243 | Intron8 | G | T |
| rs7200972 | 54036352 | Intron8 | A | G |
| rs4784338 | 54037971 | Intron8 | G | T |
| rs12600130 | 54043323 | Intron8 | C | G |
| rs17226942 | 54043514 | Intron8 | A | G |
| rs1345390 | 54044515 | Intron8 | C | T |
| rs2111118 | 54049153 | Intron8 | C | T |
| rs17227068 | 54049205 | Intron8 | A | G |
| rs2111116 | 54049252 | Intron8 | A | G |
| rs8043737 | 54049665 | Intron8 | C | T |
| rs1861554 | 54049767 | Intron8 | A | G |
| rs7194243 | 54056159 | Intron8 | C | T |
| rs860713 | 54069465 | Intron8 | A | G |
| rs2192872 | 54074627 | Intron8 | C | T |
| rs2689249 | 54082370 | Intron8 | A | G |
| rs16952906 | 54083167 | Intron8 | C | T |
| rs11076015 | 54086580 | Intron8 | C | T |
| rs2540781 | 54087859 | Intron8 | A | C |
| rs7187423 | 54090495 | Intron8 | A | G |
| rs12447427 | 54090589 | Intron8 | A | G |
| rs1558687 | 54095865 | Intron8 | C | T |
| rs2689247 | 54097159 | Intron8 | C | T |
| rs2689246 | 54097177 | Intron8 | C | T |
| rs2540784 | 54097334 | Intron8 | C | G |
| rs16952951 | 54099427 | Intron8 | A | G |
| rs16952955 | 54099469 | Intron8 | A | C |
| rs2075204 | 54100914 | Intron8 | A | G |
| rs12600060 | 54101461 | Intron8 | G | T |
| rs1420318 | 54102766 | Intron8 | A | G |
| rs2540766 | 54105971 | Intron8 | A | G |
| rs2540769 | 54107962 | Intron8 | A | C |
| rs2665275 | 54108123 | Intron8 | C | T |
| rs12599672 | 54116076 | Intron8 | A | T |
| rs3928987 | 54117511 | Intron8 | A | G |
| rs1876942 | 54118435 | Intron8 | A | C |
| rs11076017 | 54120384 | Intron8 | C | T |
| rs697769 | 54121747 | Intron8 | A | G |
| rs708254 | 54123389 | Intron8 | A | G |
| rs11863548 | 54123512 | Intron8 | A | G |
| rs708251 | 54124896 | Intron8 | A | T |
| rs2665272 | 54126617 | Intron8 | C | T |
| rs16953047 | 54130170 | Intron8 | G | T |
| rs12927155 | 54133800 | Intron8 | C | T |
| rs12445828 | 54134802 | Intron8 | C | T |
| rs2540775 | 54137259 | Intron8 | A | G |
| rs2540776 | 54137862 | Intron8 | A | G |
| rs2689269 | 54138564 | Intron8 | A | G |
